# A stepped wedge trial of efficacy and scalability of a virtual clinical pharmacy service (VCPS) in rural and remote NSW health facilities

**DOI:** 10.1186/s12913-020-05229-y

**Published:** 2020-05-04

**Authors:** Julaine Allan, Shannon Nott, Brett Chambers, Ged Hawthorn, Alice Munro, Chris Doran, Chris Oldmeadow, Clare Coleman, Teesta Saksena

**Affiliations:** 1 School of Health and Society, University of Wollongong, Wollongong, Australia; 2 Western NSW Local Health District, Dubbo, Australia; 3 Central Queensland University, Brisbane, Australia; 4 Hunter Medical Research Institute, Newcastle, Australia

**Keywords:** Clinical pharmacy, Virtual healthcare, Telehealth, Rural, Remote, Hospital, Stepped wedge, Patient experience

## Abstract

**Background:**

Medication errors are a leading cause of mortality and morbidity. Clinical pharmacy services provided in hospital can reduce medication errors and medication related harm. However, few rural or remote hospitals in Australia have a clinical pharmacy service. This study will evaluate a virtual clinical pharmacy service (VCPS) provided via telehealth to eight rural and remote hospitals in NSW, Australia.

**Methods:**

A stepped wedge cluster randomised trial design will use routinely collected data from patients’ electronic medical records (*n* = 2080) to evaluate the VCPS at eight facilities. The sequence of steps is randomised, allowing for control of potential confounding temporal trends. Primary outcomes are number of medication reconciliations completed on admission and discharge. Secondary outcomes are length of stay, falls and 28 day readmissions. A cost-effectiveness analysis (CEA) and cost-benefit analysis (CBA) will be conducted. The CEA will answer the question of whether the VCPS is more cost-effective compared to treatment as usual; the CBA will consider the rate of return on investing in the VCPS. A patient experience measure (*n* = 500) and medication adherence questionnaire (*n* = 100 pre and post) will also be used to identify patient responses to the virtual service. Focus groups will investigate implementation from hospital staff perspectives at each site.

Analyses of routine data will comprise generalised linear mixed models. Descriptive statistical analysis will summarise patient experience responses. Differences in medication adherence will be compared using linear regression models. Thematic analysis of focus groups will identify barriers and facilitators to VCPS implementation.

**Discussion:**

We aim to demonstrate the effectiveness of virtual pharmacy interventions for rural populations, and inform best practice for using virtual healthcare to improve access to pharmacy services. It is widely recognised that clinical pharmacists are best placed to reduce medication errors. However, pharmacy services are limited in rural and remote hospitals. This project will provide evidence about ways in which the benefits of hospital pharmacists can be maximised utilising telehealth technology. If successful, this project can provide a model for pharmacy delivery in rural and remote locations.

**Trial registration:**

Australian New Zealand Clinical Trials Registry (ANZCTR) -ACTRN12619001757101 Prospectively registered on 11 December 2019. Record available from: https://www.anzctr.org.au/Trial/Registration/TrialReview.aspx?id=378878&isReview=true

## Background

Globally, medication errors are a leading cause of avoidable morbidity and mortality with an estimated cost of $42 billion annually [1]. Hospital pharmacists play a key role in reducing medication errors through medication reconciliation [2–5]. Medication reconciliation (Med Rec) is the process of ensuring that patients receive prescribed medicines and that accurate, current and comprehensive medicines information follows them at all transfers of care. In hospitals, Med Rec has been reliably found to improve medication safety [6–11] and has been identified as an international priority by the World Health Organization [2, 6, 12].

In Australia medication safety is addressed through Standard 4 of the National Safety & Quality Health Service Standards (NSQHS) which includes safe prescribing, dispensing, administering and monitoring of appropriate medicines to informed patients [13]. However, medication-related incidents are the second most frequently reported incidents in NSW hospitals [14]. Consequently, improved medication management is a high priority, particularly in rural and remote settings where clinical pharmacy services are limited.

Australian research indicates that patients’ problems understanding and using their medications increases the risk of readmission 2.3 times [8, 15]. Integrating clinical pharmacists into hospital settings improves medication management, reduces medication harm, supports patients and decreases unwarranted clinical variation [16]. However, there is limited provision of Med Rec in rural Australian hospitals due to few on-site pharmacists [17–20] consequently patients are at a higher risk for medication-related harms [21]. Virtual or telehealth pharmacy services are one option to address the lack of on-site pharmacists.

Virtual pharmacy services have been implemented across parts of rural and remote Australia, particularly Queensland, however much of the literature reports on outpatient medication reviews rather than inpatient services [16, 22–24]. A feasibility study evaluating a service providing virtual clinical pharmacy reviews for inpatients in rural and remote regions was found to be acceptable [23]. Despite this, few studies have since reviewed service efficacy, with most studies focussing on patient satisfaction [23]. The use of virtual service delivery has been demonstrated to increase accessibility to specialist care particularly for rural and remote populations including Aboriginal communities, with evidence supporting the use of virtual technologies for the management of chronic diseases [25].

Three small studies have assessed the feasibility and acceptability of virtual pharmacy services in rural and remote locations [16, 23, 24]. This study will add to existing literature to provide evidence about the feasibility and efficacy of virtual pharmacy services in relation to patient outcomes, patient experience and economic factors.

The VCPS evaluation aims to 1.) Investigate if the VCPS results in a detection of preventable medication harms, reductions of readmission, falls and length of stay; 2.) Identify if the VCPS is cost-effective; and 3.) Assess if the VCPS is perceived to be an acceptable service for patients and clinicians.

Western NSW Local Health District (WNSWLHD) and Far West Local Health District (FWLHD) comprise some of the most remote regions in NSW, Australia. The two districts cover almost 450,000km^2^ with a population of 300,000 people. Services are provided by a combination of rural referral hospitals, rural hospitals, multipurpose facilities and nurse-only remote clinics. Onsite clinical pharmacy services are provided to only seven of thirty eight facilities, resulting in noncompliance with Standard 4 of the National Safety & Quality Health Service Standards (NSQHS) [13]. Existing telehealth infrastructure along with recent roll out of electronic medication management (eMeds) in the electronic medical record (eMR), has provided the infrastructure for a virtual clinical pharmacy service (VCPS).

## Methods/design

**Fig. 1:**
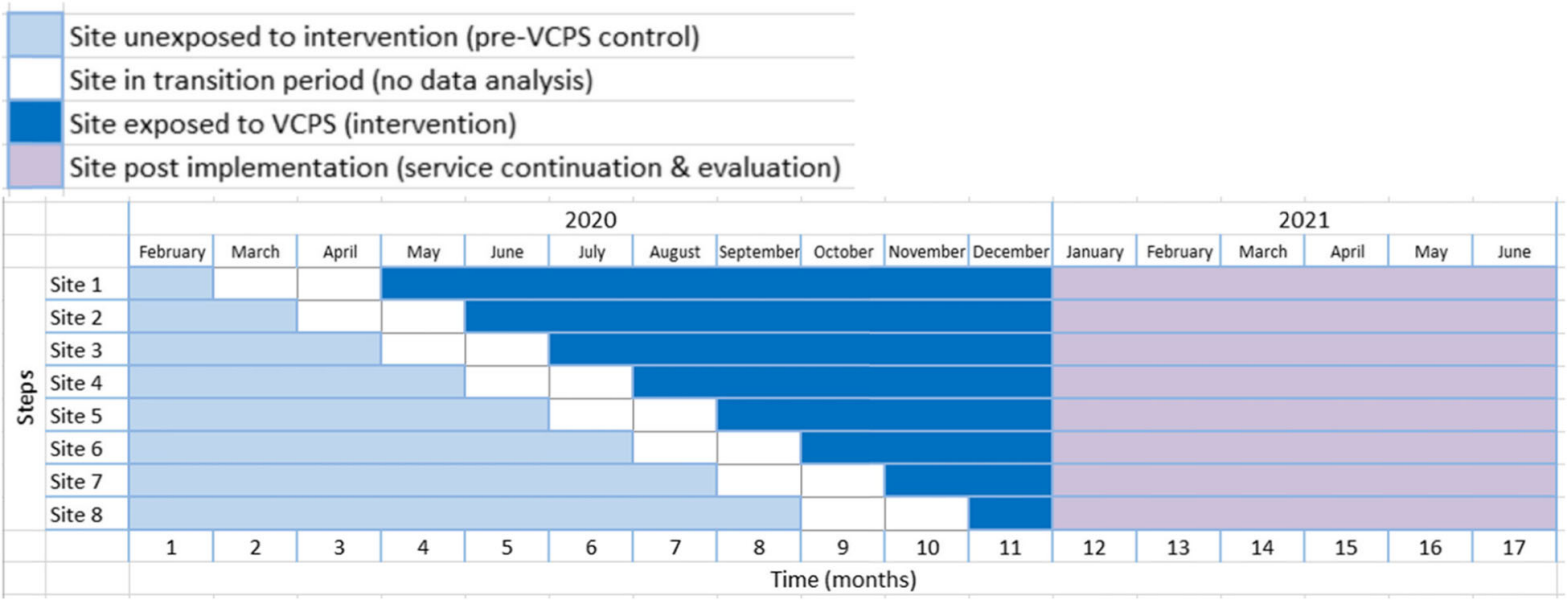
VCPS stepped wedge cluster randomised control trial design. There are a total of 8 steps, with each step being one month apart. The light blue represent sites unexposed to the intervention (control). White represents sites where the service is being introduced and no data will be used in the analysis. Dark blue represents sites exposed to the VCPS, and purple the service continuation and evaluation

The VCPS evaluation will employ a stepped wedge cluster randomised trial design, where the intervention is sequentially implemented at eight facilities. The ‘steps’ are the order in which these sites cross-over from the control condition (pre-VCPS) to the intervention condition (VCPS). The sequence of the steps is randomised, allowing for control of potential confounding temporal trends. The cross-over will occur across 8 waves or steps (one site per step), each 1 month apart with a 2 month ‘in-transition’ period (see Fig. 1).

The virtual pharmacy service will be delivered in six rural NSW hospitals that do not have an on-site pharmacist but are using electronic medication management and patient record systems and have more than twenty inpatient admissions per month; and two remote hospitals that have between seven and thirteen admissions per month and fit the other inclusion criteria. All adult patients of emergency departments and inpatient wards of the participating hospitals will be included in the study. The order of the site implementation will be determined by computer-generated random numbers.

The intervention will consist of utilising the eMR, eMeds and a wireless teleconferencing cart with two–way audio and visual called a ‘Wallie’ to provide clinical pharmacy services to study sites. Virtual Pharmacists will work 8.30 am to 4.30 pm, Monday-Friday of each week over the study period where it is anticipated they will see up to ten patients per pharmacist per day.

Virtual clinical pharmacists will conduct Med Rec on admission, discharge or transfer and document a medication history in eMeds. A summary of the Med Rec and any identified issues will be documented on the eMR Medication Management Plan. To ensure continuity of medication management on discharge back to the community, the pharmacist will provide an updated medication list on discharge. The virtual pharmacist will provide comprehensive information to the patient or carer to enable safe and effective use of the medications. This will be tailored to suit the patient’s individual circumstances and may be in the form of verbal instructions, demonstration, education, a medication list, written advice or a consumer medicines information sheet. Pharmacists will also undertake clinical medication review for inpatients of the facilities and provide medication advice to Doctors and Nurses. Any harms or clinical incidents arising from the VCPS will be addressed using the NSW Health Incident Management System (IMS) and investigations will be conducted according to clinical governance processes.

Routinely collected, de-identified health data will be analysed pre and post VCPS (see Table 1). Data will be collected from the electronic medical record and Health Intelligence Unit (HIU) reports. A waiver of consent has been granted for this de-identified data. Patients receiving the service will be invited by nursing staff at their facility to complete a patient experience measure and a medication non-adherence questionnaire [26]. Staff at participating hospitals will be invited by email from the research team to a focus group about their experience of the virtual pharmacy service. Participation will be voluntary and written consent required.

**Table 1:** Measures and routinely collected data source for VCPS evaluation

Outcome Measure	Data Source	Level of Data
% medication reconciliation on admission	eMR Report EM002	Facility
% medication reconciliation on discharge	eMR Report EM002	Facility
VTE Prophylaxis rates	Audit Office report	Facility
Antimicrobial usage	iPharmacy Dispensing Software	Facility
% medication list on discharge	Custom eMR report	Patient
28 day readmission	HIU report	Facility
28 day readmission Aboriginal patients	HIU report	Facility
Length of stay	HIU report	Facility
Length of stay Aboriginal patients	HIU report	Facility
Falls	HIU report	Facility
Detection of preventable medication errors	HIU report	Facility
Economic analysis (cost effectiveness) & scalability	HIU reports	Facility
**Process Measure**	**Data Source**	**Level of Data**
Number of medication reconciliation completed	eMR report EM002	Facility
Number of medication lists on discharge	Custom eMR report	Patient
Number of referrals/ number of referrals completed	eMR report PC011	Patient
Pharmacist Interventions	eMR DA2 Clinical Pharmacy Interventions	Patient
Time taken to undertake interventions	eMR DA2 Clinical Pharmacy Interventions	Patient
Time taken to provide education	eMR DA2 Clinical Pharmacy Interventions	Patient
Number of consultations	eMR report PC011	Patient
Uptake of pharmacy recommendations	eMR DA2 Clinical Pharmacy Interventions	Patient
Number of pharmacist AMS reviews	eMR report PC011/ eMR Report EM008	Patient

The primary outcome variables are: 1) the proportion of separations (“discharged home by the hospital”) where the medication reconciliation occurred on admission; and 2) the proportion of separations (“discharged home by the hospital”) where the medication reconciliation occurred on discharge. Secondary outcome variables include 28-day readmission, hospital length of stay (LOS), number of falls and detection of medication-related errors. Our analyses will comprise generalised linear mixed models, with fixed effects for time, period (pre vs post), and patient-level confounding variables, as well as random effects for facility and patient to model clustering/repeat measures. Analyses will exclude separations where the patient is transferred to another facility.

Sample size: Across the eight sites a referral rate of 29 patients per month is estimated yielding approximately 2088 patients over the intervention period. Assuming a baseline medication reconciliation rate on admission of 11% (a similar rate for discharge medication reconciliations), an intra-class correlation of 0.05, and an average of 29 patient separations per site per month,) there will be over 90% power to detect an absolute 10% increase in the proportion of reconciliations performed on admission and discharge, with a type 1 error rate of 2.5%.

A PREMS will be conducted with patients who have seen the virtual pharmacist. The PREM survey consists of 10 questions which will be delivered while the patient is in hospital using a tablet device and the Customer Feedback Solutions electronic system. The PREMS will be delivered electronically via Customer Feedback Systems (CFS). CFS will securely store the results and provide de-identified reports monthly.

The PREMS will be used to quantitatively assess improvements in patients’ knowledge and perceived acceptability of virtual pharmacy. The VCPS questions address patient communication of medication management during hospital admission, patient’s confidence managing medications and overall satisfaction with the VCPS.

To evaluate the acceptability of VCPS against face to face pharmacy services, PREMS will be conducted on the general medical wards at two hospitals with patients who have seen a face to face inpatient pharmacist. The general medical ward was chosen as it will have the most similar patient population to the study sites. Data will be collected from the comparison sites between May 2020 and June 2021.

All inpatients receiving the VCPS will be invited to complete the PREMS (estimated *n* = 500). All inpatients receiving an inpatient face to face pharmacy service on the medical wards at two comparison hospitals within a 3 month time period (to be negotiated with the sites) between March 2020 and June 2021 will be asked to complete the PREMS (estimated *n* = 500). A sample of this size will allow detection of differences in responses between demographic sub-groups of at least 0.3 standard deviations (i.e. a moderate effect size), with 90% power and 5% type 1 error rate. Descriptive statistical analysis will summarise patient answers, and linear regression models will be used to explore characteristics associated with the overall score using age, sex and Aboriginality as independent variables.

Medication adherence refers to whether a patient takes their medications as prescribed and is a primary determinant of treatment success. Poor adherence to medications is common in patients with chronic disease and in patients prescribed preventative medications. The 26 question Voil’s Medication non-adherence questionnaire (Voil’s) is validated to identify non-adherence behaviour in a number of chronic disease populations and scores have been show to correlate well with objective measures and clinical outcomes [26]. The measure records reasons for nonadherence. This is important in a rural and remote setting where availability of medication may be limited or the cost prohibitive. Identifying the reasons for non-adherence allows interventions to be tailored to those.

The Voil’s will be administered to patients on admission to study sites in both control and intervention periods electronically via Customer Feedback Systems (CFS). CFS will securely store the results and provide de-identified reports monthly. The Voil’s will then be repeated 4 weeks post discharge to assess changes in medication adherence due to the VCPS. A follow-up survey will be administered via text message invitation requesting the patient complete the questionnaire electronically via the Customer Feedback Systems platform. A sample of 75 patients in the pre period and 75 patients in the post period (across all sites) will give the study 85% power to detect an increase in adherence of 0.5 standard deviations (a moderate effect size), with a type 1 error rate of 5%.

A list of eligible patients that are admitted will be used as a sampling frame, and patients will be randomly selected from this frame using a computerised program, and approached to complete the Voil’s. A participation consent form is available from the corresponding author.

Characteristics of patients responding in pre and post collection periods will be compared using independent sample t-tests (for continuous variables) or chi-square tests (for categorical variables). Differences in adherence between patients in the pre period and patients in the post period will be compared using linear regression models, adjusting for patient characteristics that may have changed between the periods.

An economic analysis will consist of a cost-effectiveness analysis (CEA) and cost-benefit analysis (CBA) [27, 28]. These analyses will be conducted in accordance with international best-practice for such analyses in health care including: adoption a health sector perspective; transparent and scientific methods to identify, measure and value both costs and outcomes; modelling and uncertainty testing of input parameters; and, interpretation of results within a broader decision-making framework. The CEA will answer the question of whether the VCPS is more cost-effective than compared to treatment as usual; the CBA will consider the rate of return on investing in the VCPS. Both analyses will rely on health care utilisation data outlined in Table 1 that identifies and measures resource use associated with the VCPS including: additional pharmacist time to deliver the intervention; patient and clinician education; and, any system level changes that have occurred to facilitate the implementation of the intervention. Resource use will be collected by the virtual clinical pharmacist and stored in the patient’s clinical record on the eMR as in standard clinical practice. Change in 28 day re-admission will be used as the primary outcome in the CEA. The CBA will attempt to quantify a wider range of outcomes associated with the VCPS that can be valued in monetary terms such as LOS, the cost of treating adverse events (falls, medication-related errors) and potentially preventable admissions. Monte Carlo analysis will be used to derive 95% uncertainty intervals for costs and outcome with results presented in a scatterplot (i.e. cost-effectiveness plane). The results of the CEA will be considered in the context of other decision-making criteria such as: capacity of the intervention to reduce inequity; acceptability to stakeholders; feasibility; sustainability; and, potential for other consequences. Results of the CBA will be expressed as a ratio of benefits to costs whereby a benefit cost ratio > 1, suggests that the VCPS is a good return on investment.

A staff focus group will be held at each participating site 3 months after the beginning of the VCPS transition period. The focus group will investigate the perceived acceptability of the service for staff. All staff who work in clinical areas where the VCPS operates will be asked to participate in the focus group. Senior staff and managers’ will participate in a separate focus group to all other staff to take into account potential power relationships that may inhibit the responses of junior staff. The focus group will be held 3 months after the VCPS commences to provide adequate time for staff to be exposed to the service. The focus group discussion will explore the issues, benefits, barriers and overall acceptability of the VCPS. A participation consent form is available from the corresponding author.

A qualitative approach will be used to undertake focus groups with participating staff (*n* = 25). The focus groups will investigate implementation processes and those affected by them to understand the context of VCPS implementation [29, 30]. The focus groups will be audio-recorded, transcribed verbatim and entered into QSR NVivo. Thematic analysis will be utilised to deductively and inductively identify barriers and facilitators to the VCPS implementation. Data files and transcripts will be stored on a password protected server only accessible by the research team.

There was no patient or public involvement in the research design. Community consultation with Australian Indigenous communities in the study sites will be conducted prior to the VCPS implementation to explain the project and identify and respond to any concerns. Indigenous Australian comprise up to 60 % of the population in some sites. If community Elders do not support the pharmacy service, or the study, Indigenous people will not be included in the data collection. Indigenous research team members will conduct the consultations.

Data monitoring committee: A data monitoring committee has not been formed because there are minimal risks associated with the study. However, a project governance group has been formed that includes academic and research pharmacists, government clinical practice and telehealth specialists and local health executives. This group will meet quarterly and will be responsible for monitoring the project progress and addressing any problems that arise.

The study results will be communicated to the funding body in regular reports. The research team will also seek to publish the results in peer reviewed journals, and in newsletters and research snapshots back to the hospital sites and surrounding communities where the trial took place. There are no restrictions on publication. Authorship eligibility will follow the International Committee of Medical Journal Editors (ICMJE) guidelines. There are no plans to grant public access to the participant level dataset. The full protocol is available from ANZCTR.

## Discussion

There is good evidence that hospital based clinical pharmacy services demonstrate measurable outcomes in reducing preventable admissions to hospital, minimising medication-related harm, improving medication-related communication on transfer of care to primary care providers, empowering patients with a better understanding of their medications, improve concordance with medication management and improve evidence-based prescribing (including antimicrobial stewardship) [7, 8, 15, 21]. However, there is limited quality evidence that similar outcomes can be achieved through the provision of virtual pharmacy services. This protocol paper describes a stepped wedge clustered randomised control trial of virtual pharmacy services delivered in eight rural and remote hospitals.

If successful, this project can provide a model for pharmacy delivery in rural and remote locations. Moreover, this trial has the potential to assist with medication literacy and concordance amongst Aboriginal patients who present to participating facilities. All rural and remote Australians, including Aboriginal peoples, have a shorter life expectancy and a more significant burden of chronic disease which equates to greater polypharmacy and a higher risk of medication-related harm [31].

It is widely recognised that clinical pharmacists are best placed to reduce rates of medication error [6, 7]; however, the workforce is poorly integrated into rural and remote health facilities [17, 18]. This project will provide evidence about ways in which the proven benefits of hospital pharmacists can be maximised utilising telehealth technology. The results of this study may lead to the development of a proven model of care that could easily be scaled and implemented in other local health districts in NSW and Australia.

The pragmatic design and sample of the study also greatly increases the generalisability by: 1.) using a community sample; and 2.) using a model that mirrors routine clinical care with routine data collection to increase validity, reliability and generalisability of results to other rural and remote hospital sites.

We also acknowledge the limitations of this trial, including: variability of sites, staffing and patient populations and self-report measures of medication adherence. First, participating sites are all small rural or remote hospitals with varying numbers and skill sets of staff. While technology has been well integrated into healthcare across the region, staff may not be familiar with how to use it or there could be system failures that prevent the pharmacy service delivery.

The identified limitations highlight the challenges of running an RCT of this kind under real-world conditions (e.g. casual staff, working with a diverse population, etc.). Despite these potential limitations, this trial is one of the most methodologically rigorous trials of virtual pharmacy interventions that exists to date. We will make extensive efforts to control for any sources of potential bias, imprecision, or multiplicity of analyses. Should modifications to the protocol be required these will be updated in the ANZCTR record as soon as they occur (Appendix).

This paper describes the protocol for a stepped wedge clustered randomized controlled trial designed to evaluate the effectiveness of a virtual pharmacy service delivered in eight rural or remote hospital sites. We aim to demonstrate the effectiveness of pharmacy interventions for rural populations, and inform best practise for using virtual healthcare to improve access to pharmacy services.

## Data Availability

Not applicable to study protocol. Access to the final trial dataset in limited to the research team and they are not limited by any contractual agreements.

## Appendix

**Table:**

Data category	Information^**32**^
Primary registry and trial identifying number	Australian New Zealand Clinical Trials Registry (ANZCTR) -ACTRN12619001757101 Registered on
Date of registration in primary registry	11 December 2019.
Secondary identifying numbers	NA
Source(s) of monetary or material support	NSW Ministry of Health, Australia
Primary sponsor	NSW Ministry of Health, Australia
Secondary sponsor(s)	NA
Contact for public queries	*Brett Chambers* [+ 61,447,482,949] [brett.chambers@health.nsw.gov.au]
Contact for scientific queries	*Brett Chambers* [+ 61,447,482,949] [brett.chambers@health.nsw.gov.au]
Public title	A stepped wedge trial of efficacy and scalability of a virtual clinical pharmacy service (VCPS) in rural and remote NSW health facilities
Scientific title	*A stepped wedge trial of efficacy and scalability of a virtual clinical pharmacy service (VCPS) in rural and remote NSW health facilities*
Countries of recruitment	Australia
Health condition(s) or problem(s) studied	Clinical pharmacy services
Intervention(s)	Virtual clinical pharmacy service to rural and remote hospitals
Key inclusion and exclusion criteria	Ages eligible for study: ≥18 yearsSexes eligible for study: bothAccepts healthy volunteers: noInclusion criteria: adult patient (≥ 18 years) hospitalised in one of the study sites requiring medication reconciliation or other pharmacy serviceExclusion criteria: Outpatients, Residential Aged Care, Transitional Aged Care, Hospital in the Home
Study type	InterventionalAllocation: randomizedIntervention model: Stepped wedgeMasking: NoPrimary purpose: treatment
Date of first enrolment	April 2020
Target sample size	2088
Recruitment status	Recruiting
Primary outcome(s)	There are 2 co-primary outcome variables: 1) the proportion of separations (“discharged home by the hospital”) where the medical reconciliation occurred on admission; and 2) the proportion of separations (“discharged home by the hospital”) where the medical reconciliation occurred on discharge.
Key secondary outcomes	Secondary outcomes will include 28-day readmission, hospital length of stay (LOS), number of falls and detection of medication-related errors.
Protocol Version	Version 2.1:03 March 2020

## Abbreviations

ANZCTR: Australian and New Zealand clinical trials registry
CBA: Cost benefit analysis
CEA: Cost effectiveness analysis
CFS: Customer feedback systems
eMEDS: Electronic medication management
eMR: Electronic medical record
FWLHD: Far West local health district
HIU: Health intelligence unit
ICMJE: International committee of medical journal editors
IMS: Incident management system
LOS: Length of stay
Med Rec: Medication reconciliation
NSQHS: National safety & quality health service standards
NSW: New South Wales
PREMS: Patient reported experience measure
RCT: Randomised controlled trial
VCPS: Virtual clinical pharmacy service
WNSWLHD: Western NSW local health district
